# Ten weeks of high-intensity interval walk training is associated with reduced disease activity and improved innate immune function in older adults with rheumatoid arthritis: a pilot study

**DOI:** 10.1186/s13075-018-1624-x

**Published:** 2018-06-14

**Authors:** David B. Bartlett, Leslie H. Willis, Cris A. Slentz, Andrew Hoselton, Leslie Kelly, Janet L. Huebner, Virginia B. Kraus, Jennifer Moss, Michael J. Muehlbauer, Guillaume Spielmann, William E. Kraus, Janet M. Lord, Kim M. Huffman

**Affiliations:** 1 Duke Molecular Physiology Institute, Duke University School of Medicine, Durham, NC USA; 2 Division of Medical Oncology, Duke University School of Medicine, Durham, NC 27701 USA; 3 MRC-ARUK Centre for Musculoskeletal Ageing Research, Institute of Inflammation and Ageing, University of Birmingham, Birmingham, UK; 4 Department of Kinesiology, Louisiana State University, Baton Rouge, LA USA; 5 NIHR Birmingham Biomedical Research Centre in Inflammation, University Hospital Birmingham, Birmingham, UK

**Keywords:** Rheumatoid arthritis, Disease activity, Innate immunity, Inflammation, High-intensity interval exercise

## Abstract

**Background:**

Rheumatoid arthritis (RA) is a chronic inflammatory disease in which adults have significant joint issues leading to poor health. Poor health is compounded by many factors, including exercise avoidance and increased risk of opportunistic infection. Exercise training can improve the health of patients with RA and potentially improve immune function; however, information on the effects of high-intensity interval training (HIIT) in RA is limited. We sought to determine whether 10 weeks of a walking-based HIIT program would be associated with health improvements as measured by disease activity and aerobic fitness. Further, we assessed whether HIIT was associated with improved immune function, specifically antimicrobial/bacterial functions of neutrophils and monocytes.

**Methods:**

Twelve physically inactive adults aged 64 ± 7 years with either seropositive or radiographically proven (bone erosions) RA completed 10 weeks of high-intensity interval walking. Training consisted of 3 × 30-minute sessions/week of ten ≥ 60-second intervals of high intensity (80–90% VO_2reserve_) separated by similar bouts of lower-intensity intervals (50–60% VO_2reserve_). Pre- and postintervention assessments included aerobic and physical function; disease activity as measured by Disease Activity score in 28 joints (DAS28), self-perceived health, C-reactive protein (CRP), and erythrocyte sedimentation rate (ESR); plasma interleukin (IL)-1β, IL-6, chemokine (C-X-C motif) ligand (CXCL)-8, IL-10, and tumor necrosis factor (TNF)-α concentrations; and neutrophil and monocyte phenotypes and functions.

**Results:**

Despite minimal body composition change, cardiorespiratory fitness increased by 9% (change in both relative and absolute aerobic capacity; *p* < 0.001), and resting blood pressure and heart rate were both reduced (both *p* < 0.05). Postintervention disease activity was reduced by 38% (DAS28; *p* = 0.001) with significant reductions in ESR and swollen joints as well as improved self-perceived health. Neutrophil migration toward CXCL-8 (*p* = 0.003), phagocytosis of *Escherichia coli* (*p* = 0.03), and ROS production (*p* < 0.001) all increased following training. The frequency of cluster of differentiation 14-positive (CD14^+^)/CD16^+^ monocytes was reduced (*p* = 0.002), with both nonclassical (CD14^dim^/CD16^bright^) and intermediate (CD14^bright^/CD16^positive^) monocytes being reduced (both *p* < 0.05). Following training, the cell surface expression of intermediate monocyte Toll-like receptor 2 (TLR2), TLR4, and HLA-DR was reduced (all *p* < 0.05), and monocyte phagocytosis of *E. coli* increased (*p* = 0.02). No changes were observed for inflammatory markers IL-1β, IL-6, CXCL-8, IL-10, CRP, or TNF-α.

**Conclusions:**

We report for the first time, to our knowledge, that a high-intensity interval walking protocol in older adults with stable RA is associated with reduced disease activity, improved cardiovascular fitness, and improved innate immune functions, indicative of reduced infection risk and inflammatory potential. Importantly, the exercise program was well tolerated by these patients.

**Trial registration:**

ClinicalTrials.gov, NCT02528344. Registered on 19 August 2015.

## Background

Rheumatoid arthritis (RA) is a chronic inflammatory disease characterized by swollen and painful joints, synovial inflammation, deformation of cartilage and bone structures, and a dysfunctional autoreactive immune system [1]. Highly differentiated, apoptosis-resistant innate immune cells characterize the pathology of RA; neutrophils dominate synovial fluid, and monocyte/macrophages infiltrate the expanded synovium. The proinflammatory milieu augments disease pathology by extending the lifespan of these cells and ensuring their retention in the joint. In the peripheral blood, immune cells show evidence of accelerated immune aging, with patients displaying a level of cell senescence/exhaustion suggestive of an adult several years older [2].

As such, in patients with RA, dysfunctional peripheral blood neutrophil migration promotes both nonspecific tissue damage and poor resolution of infection [3, 4]. Additionally, neutrophil phagocytic capacity is reduced, leading to increased risk of infections [5]. Although neutrophils in the joint produce more ROS [6], in the peripheral blood neutrophil ROS are similar or reduced compared with those of healthy control subjects [5, 7]. Furthermore, as compared with healthy individuals, peripheral blood monocyte/macrophages in patients with RA are characterized by an increased frequency of proinflammatory monocytes (cluster of differentiation 14-positive [CD14^+^]/CD16^+^) in the circulation [8–11]. These cells play a major pathogenic role in RA, and reduction of their frequency may be of benefit to patients with RA [12, 13]. Further, blood monocytes have reduced phagocytic and antigen presentation capacity in RA, adding to the risk of infection associated with poor neutrophil function [14]. As such, RA is associated with an impaired peripheral immune system compounded by immunosuppressive medications, leading to an increased risk of opportunistic infections, both bacterial and viral [15]. Therefore, identifying ways to reduce these risks and improve antibacterial immunity is critical to improving the health and quality of life of adults with RA.

In addition to a dysfunctional immune system, people with RA are often severely physically inactive owing to issues with pain and fatigue; inactivity leads to poor muscle function, low cardiorespiratory fitness, and worsened disability [16]. Current U.S. health guidelines and the American College of Rheumatology both suggest resistance exercises to improve muscle strength and quality and aerobic exercises to improve cardiorespiratory fitness. Although it is unclear whether any form of exercise can improve disease activity scores, there is a general consensus that short- and long-duration aerobic and resistance exercise programs do not worsen them [17].

In persons without RA, one of the underlying mechanisms by which exercise promotes improved health outcomes is through its anti-inflammatory effects [18, 19]. Anti-inflammatory effects are promoted by a range of intrinsically related factors. Alongside improved metabolic features associated with muscle and adipose tissue, improvements in peripheral primary immune function have the potential to limit inflammatory insult [18, 20–23]. Although it is unclear which factor has the largest impact, each has individual health benefits. We have recently shown that in older healthy adults, better directional neutrophil migration toward the chemokine (C-X-C motif) ligand (CXCL-8) is associated with increased physical activity, suggesting that exercise training might improve neutrophil migratory functions [20]. Blood monocyte phenotype can be modified by aerobic and resistance exercise training, suggesting that exercise training may improve the dysregulated monocyte/macrophage inflammatory potential in patients with RA [24–26]. Therefore, exercise programs that improve cardiovascular fitness and muscle function may improve long-term health in patients with RA by modifying immune function.

High-intensity interval training (HIIT) is an exercise activity that may improve the health of patients with RA. In persons without RA, HIIT is equivalent or superior to moderate-intensity continuous training at improving cardiorespiratory fitness when matched for energy expenditure or time spent in the activity [25, 27]. HIIT also improves immune cell functions, specifically peripheral blood neutrophil and monocyte functions [25, 28, 29]. Critically, HIIT does not increase concentrations of systemic inflammatory cytokines, which may be detrimental to patients with RA [25]. However, few studies have assessed HIIT in RA to modify disease activity, cardiorespiratory fitness, physical function, or immune activity. Those which have suggest that HIIT is both safe and well tolerated and can rapidly increase cardiorespiratory fitness and muscle strength and also improve joint health [30–32].

Therefore, the purpose of this pilot study was to determine the efficacy of 10 weeks of HIIT walking for improving disease activity and aerobic capacity in older (> 55 years) adults with low to moderate RA. Additionally, we aimed to assess the effects of HIIT walking on peripheral blood neutrophil and monocyte antibacterial function and systemic inflammatory cytokine concentrations, as well as to generate effect size estimates for a larger clinical trial. We hypothesized that HIIT would increase aerobic capacity and reduce disease activity.

## Methods

### Participants

Twelve sedentary participants (11 women and 1 man) aged 64 ± 7 years and with confirmed and stable RA were recruited for the study. Participants were either seropositive or had radiographic joint erosions of finger joints, met the 1987 American College of Rheumatology criteria for RA [33], had no medication changes in the previous 3 months, and were using prednisone ≤ 5 mg/d. Exclusions were known diabetes mellitus or cardiovascular disease and an inability to walk unaided on a treadmill. All participants gave written informed consent, and the study was approved by the Duke University Medical Center Institutional Review Board (IRB no. Pro00064057).

### Exercise training

Exercise training consisted of 10 weeks of 3 × 30-minute sessions per week of supervised treadmill walking. In aggregate, participants completed 99% of prescribed exercise sessions. Exercise intensities were determined from a cardiorespiratory fitness test. For exercise prescription, VO_2_ reserve was chosen and calculated as previously described [34]. Participants were given between three and six sessions to become accustomed to the exercise (30- to 45-second intervals at target heart rates; total time, 20 minutes). Exercise consisted of a 5-minute warm-up and 5-minute cool-down as part of the total session. Intervals were designed to elicit a heart rate corresponding to 80–90% of VO_2_ reserve (high intensity = actual heart rate percentage of 85 ± 5%) and 50–60% VO_2_ reserve (active recovery). Speeds did not exceed walking pace (range, 1–4.6 mph), and if heart rate was not achieved by walking speed, gradient (range, 2–15%) was added to increase heart rate. High-intensity intervals were between 60 and 90 seconds, followed by active recovery intervals of a similar duration. Rather than controlling each session for energy expenditure, total intervals per session were adjusted so that the exercise duration per session was 30 minutes. Ratings of perceived exertion were detailed at the end of each high-intensity interval bout.

### Assessment of disease activity

All participants were examined by a rheumatologist using previously described methods for determining disease activity using the Disease Activity Score in 28 joints (DAS28) [35]. This score includes assessment of the number of swollen and tender joints, a visual analogue scale for general health, and C-reactive protein (CRP) concentration or erythrocyte sedimentation rate (ESR) (DAS28_CRP_ and DAS28_ESR_, respectively).

### Fitness, function, and body composition

Exercise treadmill testing was used to assess cardiorespiratory fitness. Aerobic capacity (VO_2peak_) was determined by a graded maximal treadmill test starting at 2 mph/0% grade and then increasing speed and/or grade such that the metabolic demand increased at approximately 3.5 ml/kg/min until volitional exhaustion. Twelve-lead electrocardiogram, ventilation, and gas exchange were continuously assessed and recorded as 15-second averages using a Parvo Metabolic Cart (Parvo Medics, Sandy, UT, USA). Highest 15-second values were used for determination of VO_2peak_, and a valid test was confirmed by either a respiratory exchange ratio (RER) > 1.1 (mean RER, 1.2 ± 0.1 at both times) or a rating of perceived exertion ≥ 17. Body composition was assessed according to Siri’s three-compartmental model [36]. Body weight, fat mass, and lean mass were determined by air displacement plethysmography (BOD POD System; COSMED, Rome, Italy) [37]. Resting blood pressure and heart rate were taken following 15 minutes of sitting quietly. Each participant also completed a short battery of physical functioning tests that are indicative of health and frailty in older adults [38–40]. Grip strength was assessed by dynamometry in both hands in triplicate; the best score was taken. Timed Up and Go (TUG), Berg Balance Scale, 30-second chair stands, and 400-m walk tests were all supervised by a trained exercise physiologist. All tests were completed before training and at least 24 hours after the last exercise session.

### Physical activity and health questionnaires

During their fasting blood draw visits (*see below*) at baseline and following the intervention, participants completed standardized validated physical activity and health questionnaires. Physical activity questionnaires were used to determine that participants were not engaging in more physical activity than what was prescribed in the exercise program. Questionnaires used included the Incidental and Planned Exercise Questionnaire (IPEQ), the Stanford Brief Activity Survey (SBAS), the Hospital Anxiety and Depression Scale (HADS), a visual analogue scale for pain (VAS), and the Health Assessment Questionnaire Disability Index (HAQ-DI).

### Fasting glucose and insulin

Blood was obtained following a 10-hour overnight fast both before training and between 16 and 24 hours after the last exercise bout. Glucose was measured with a YSI Biochemistry Analyzer (YSI Inc., Yellow Springs, OH, USA), and insulin was measured by enzyme-linked immunosorbent assay (ELISA) according to the manufacturer’s guidelines (Thermo Fisher Scientific, Waltham, MA, USA); both were measured in duplicate. Hemoglobin A1C (HbA1c) values were obtained through a commercial clinical analysis company (LabCorp, Burlington, NC, USA). The homeostasis model assessment of insulin resistance (HOMA-IR) was calculated as described previously using the HOMA2-IR online calculator [41, 42].

### Immune cell isolation

Complete blood count differentials were measured using a commercial clinical analysis company (LabCorp). Neutrophils were isolated from heparin-treated blood following 2% dextran sedimentation and separation on a discontinuous Percoll gradient as previously described [20]. Neutrophil purity and viability were determined by Giemsa staining (Diff-Quik; Gentaur Europe, Kampenhout, Belgium) and trypan blue exclusion, respectively. Neutrophil purity and viability were consistently ≥ 95%. Neutrophils were resuspended at 3 × 10^6^ cells/ml in either RPMI 1640 (+calcium, +magnesium: RPMI) medium only (Sigma-Aldrich, St. Louis, MO, USA), RPMI 1640 containing 0.15% bovine serum albumin (BSA; RPMI-BSA; Sigma-Aldrich) or HBSS+/+ (+ calcium, + magnesium) medium, depending on the assay.

Peripheral blood mononuclear cells (PBMCs) were isolated by Ficoll-Paque (GE Healthcare Life Sciences, Marlborough, MA, USA) density gradient centrifugation. Briefly, blood was diluted 1:1 with RPMI 1640 and layered over the Ficoll before centrifugation at 400 × *g* for 30 minutes at room temperature. The mononuclear cell layer was removed and washed twice before being resuspended at 1 × 10^6^ cells/ml in RPMI + 1% BSA.

### Neutrophil migration

Neutrophil migratory dynamics were assessed using an Insall chamber (Weber Scientific International Ltd., Teddington, UK) as previously described [4, 43]. Briefly, coverslips were coated with 7.5% culture-tested BSA (Sigma-Aldrich), and neutrophils were suspended in RMPI-BSA adhered to this surface for 20 minutes at room temperature. The use of this coating of BSA has previously been shown to mimic the ligand for CD11b and CD18, intercellular adhesion molecule (ICAM)-1 [44]. The coverslip was then inverted on the Insall chamber before addition of buffer (RPMI) alone as a control or buffer containing 100 nM CXCL-8 (R&D Systems, Minneapolis, MN, USA).

Neutrophil migration was monitored using a Zeiss Axiovert 100 inverted microscope (Carl Zeiss Microscopy, Buffalo Grove, IL, USA) fitted with a Hamamatsu ORCA 100 digital camera (Hamamatsu, Japan). Time-lapse recordings and calculations of neutrophil migratory dynamics were performed as previously described [4]. Briefly, the Insall chamber allows the formation of stable chemoattractant gradients, with defined, consistent direction in the *y* direction for each experiment [43]. Only distance traveled in the *y* direction over time was included in calculations of chemotaxis. Migration was assessed using three parameters: average cell speed (μm/min) of movement toward the chemokine (termed *chemokinesis*), average velocity (μm/min) of cells (termed *chemotaxis*), and accuracy of movement (termed *chemotactic index*). Chemotactic index is expressed in a comparative scale and arbitrary units (a.u.) ranging from − 1 to + 1. Movement directly toward the chemoattractant is + 1, whereas movement directly away is − 1.

Recordings lasted 20 minutes per experiment, with 20 slides captured using OpenLab software (Improvision, Coventry, UK). The Java software ImageJ (Wayne Rasband, National Institutes of Health, Bethesda, MD, USA) was used to analyze cell tracks. All analyses were carried out by a single analyst blinded to subject group and cell conditions.

### Bacterial phagocytosis

Phagocytosis of opsonized fluorescein isothiocyanate (FITC)-labeled *Escherichia coli* (Thermo Fisher Scientific) was assessed in whole blood as previously described [20]. Briefly, phagocytosis was assessed in heparin-treated whole blood incubated for 10 minutes at 4 °C (control: no phagocytosis) or 37 °C (test) with FITC-labeled *E. coli*. Phagocytosis was halted by the addition of cold PBS, whereas cell surface-bound FITC was quenched by addition of 1% trypan blue solution. Unbound free bacteria were removed by washing in PBS, then erythrocytes were lysed and leukocytes were fixed using 1% fix/lyse solution (Thermo Fisher Scientific). Cell DNA was counterstained by addition of propidium iodide (PI) in order to gate on immune cells before flow cytometry was performed, and 10,000 neutrophils were acquired. Median fluorescence intensity (MFI) of the control was subtracted from the test to give a final phagocytic value. The MFI corresponds to a cell-by-cell “amount’ or phagocytic capacity of *E. coli*.

### Neutrophil ROS production

ROS generation was assessed by luminol-amplified chemiluminescence as previously described [45]. Briefly, resting neutrophils [1 × 10^5^ in HBSS+/+ (+ calcium + magnesium)] were dispensed into a 96-well, white, flat-bottomed plate (Corning, Corning, NY, USA) containing 1 μM luminol (pH 7.3; Sigma-Aldrich). Cells were stimulated with 25 nM phorbol 12-myristate 13-acetate (PMA) (test) or HBSS+/+ [46] and immediately assessed for ROS generation at 1-minute intervals for 60 minutes using an Infinite 200 PRO plate reader (Tecan Life Sciences, Männedorf, Switzerland). Experiments were performed in triplicate, with ROS production measured as relative light units and calculated as the AUC.

### Measurement of immune cell surface receptor expression

Neutrophil surface receptor expression was assessed in whole blood. Briefly, 100 μl of heparin-treated blood was dispensed into 5-ml tubes and stored at 4 °C in the dark. Cells were stained with anti-CXCR2-phycoerythrin (anti-CXCR2-PE, clone 5E8-C7-F10; Thermo Fisher Scientific), anti-CD16-FITC (clone 3G8; BD Biosciences, San Jose, CA, USA), anti-CD11b-allophycocyanin (anti-CD11b-APC, clone ICRF44; BD Biosciences), anti-CD18-PE (clone 6.7; BD Biosciences), anti-Toll-like receptor 2 (TLR2)-Alexa Fluor 647 (clone 11G7; BD Biosciences), or anti-TLR4-APC (clone HTA-125; Thermo Fisher Scientific), or their relevant concentration-matched isotype controls for 60 minutes on ice in the dark. Following incubation, cells were washed twice in cold PBS, and erythrocytes were lysed and leukocytes were fixed using 1% fix/lyse solution (Thermo Fisher Scientific). Following fixing, cells were washed twice and resuspended in 300 μl of PBS for analysis by flow cytometry.

Monocyte surface receptor expression was assessed on freshly isolated PBMCs (1 × 10^5^ cells/ml). Cells were stained with CD14-Pacific Blue (clone TuK4; Thermo Fisher Scientific), CD16-FITC, TLR2-Alexa Fluor 647, TLR4-PE, anti-HLA-DR-PE-CF594 (clone G46-6; BD Biosciences), or relevant isotype controls for 30 minutes at 4 °C in the dark. Postincubation cells were washed twice in PBS/1% BSA, resuspended in 300 μl of PBS/1% BSA, and transferred to polypropylene fluorescence-activated cell sorting tubes for analysis by flow cytometry.

All flow cytometric analyses were conducted on a BD FACSCanto II (BD Biosciences) flow cytometer equipped with three lasers using the Duke Cancer Institute Core Facility, which maintained daily quality controls of the machine. Ten thousand neutrophils and 5000 monocytes were acquired for analysis. Data were analyzed using FCS Express 6 (De Novo Software, Glendale, CA, USA).

### Plasma analyses

Samples were processed immediately for plasma and immune cell isolation, and relevant samples were stored at − 80 °C until analysis. All plasma analyses were completed by the Core Facilities within the Duke Molecular Physiology Institute. Plasma samples obtained following an overnight fast were analyzed for five cytokines and one acute-phase protein: CXCL-8, IL-6, IL-10, TNF-α, IL-1β, and C-reactive protein (CRP). Concentrations of cytokines were determined in duplicate using a human proinflammatory 5-plex sandwich immunoassay according to the manufacturer’s instructions (Meso Scale Discovery, Rockville, MD, USA). High-sensitivity CRP was measured in duplicate using a commercially available ELISA (IBL International, Hamburg, Germany). The lower limits of detection (LLODs) were as follows: CXCL-8 (0.08 pg/ml), IL-6 (0.11 pg/ml), IL-10 (0.05 pg/ml), TNF-α (0.09 pg/ml), IL-1β (0.03 pg/ml), and CRP (0.02 mg/L). All samples had concentrations greater than the LLOD, with the exception of IL-1β with 77% of samples above the LLOD. Plasma concentrations of nonesterified fatty acid (NEFA) were assessed by an enzymatic colorimetric assay on a UniCel DxC600 Analyzer using the manufacturer’s guidelines (Beckman Coulter Life Sciences, Indianapolis, IN, USA).

#### Statistics

All analyses were conducted using IBM SPSS Statistics version 23.0 software (IBM, Armonk, NY, USA), and all data are presented as mean ± SD unless otherwise stated. The primary outcome of the study was change in disease activity, and the secondary outcome was change in VO_2peak_. We initially powered the study to detect a 5% change in VO_2peak_ based on preliminary data in diabetic adults as in our previous studies [25]. The sample size for change in VO_2peak_ was five participants. Following completion of our analysis of these five participants, we powered the remainder of the study on change in disease activity. Sample size calculations suggested a total of 12 participants would be needed to detect a significant 16% reduction (80% power) in disease activity. Normality was assessed using Kolmogorov-Smirnov analysis; natural logarithmic transformation of distributed variables violating normality was completed. Pairwise comparisons of variables were completed using paired *t* tests. Bivariate correlations were conducted between changes in fitness, body composition, inflammatory markers, and immune functions to tease out associations. Statistical significance was accepted as *p* ≤ 0.05.

## Results

### HIIT effects on health and RA disease activity

Information on clinical characteristics, disease activity, and medication use is shown in Table 1. Participants were receiving a range of rheumatic medications, including immunosuppressive, anti-inflammatory, and steroidal drugs, and these medications were maintained during the study. Mean arterial blood pressure (*p* = 0.044), resting heart rate (*p* = 0.009), and feelings of depression (HADS questionnaire; *p* = 0.031) were all reduced following training. Disease activity improved by a clinically significant 38%, corresponding to a mean disease activity reduction from moderate to low (both *p* = 0.001). There was a mean 58% reduction in ESR (*p* = 0.02), but no changes for CRP (*p* > 0.05). Reductions in disease activity were mediated primarily by small reductions in the numbers of swollen and tender joints, improved global health, and in the case of DAS28_ESR_ owing to a significant reduction in ESR. There were no HIIT-induced changes in body composition, self-reported disability (HAQ-DI), pain, or anxiety (all *p* > 0.05).

**Table 1:** Before and after high-intensity interval training values, CIs, and effect sizes for clinical characteristics, health and disease activity, and medication use

	Pre-HIIT	Post-HIIT	95% CI (lower, upper)	*p* Value	Effect size (*d*)
Age, yr	64 ± 7				
Sex, M/F	1/11				
Clinical characteristics					
BMI, kg/m^2^	27.4 ± 9.3	27.7 ± 9.8	(− 0.6, 0.1)	0.191	0.40
Body fat, %	36.6 ± 11.6	37.3 ± 11.2	(− 2.1, 0.7)	0.294	0.32
Blood pressure, mmHg					
Systolic	136 ± 14	127 ± 14	(− 0.8, 17.3)	0.070	0.58
Diastolic	73 ± 12	69 ± 8	(− 2.7, 8.1)	0.064	0.60
Mean arterial pressure	94 ± 11	89 ± 9	(0.2, 10.3)	0.044	0.66
Resting heart rate, beats/min	69 ± 8	64 ± 8	(1.5, 8.3)	0.009	0.91
HbA1c, %	5.6 ± 0.5	5.6 ± 0.4	(− 0.1, 0.2)	0.898	0.04
Health and disease activity					
HAQ-DI	0.42 ± 0.35	0.39 ± 0.47	(− 0.1, 0.2)	0.614	0.16
Pain	28.7 ± 29.8	20.3 ± 21.5	(− 7.3, 24.1)	0.262	0.34
Anxiety	4.1 ± 2.6	3.5 ± 2.8	(− 0.9, 2.0)	0.393	0.25
Depression	3.8 ± 2.5	2.4 ± 1.9	(0.1, 2.5)	0.031	0.71
Swollen joints, *n*	4.3 ± 3.8	1.9 ± 1.7	(0.6, 4.2)	0.013	0.85
Tender joints, *n*	4.3 ± 7	1.9 ± 2.8	(− 0.8, 5.4)	0.062	0.48
Global health, mm	31.5 ± 15.7	22.6 ± 19.2	(0.6, 17.2)	0.037	0.85
DAS28_ESR_	3.1 ± 1.6	2.3 ± 1.2	(0.4, 1.2)	0.001	1.23
DAS28_CRP_	3.1 ± 1.2	2.4 ± 1	(0.3, 1.0)	0.001	1.26
ESR, mm/h	10.5 ± 11.9	7 ± 8.8	(0.04, 6.5)	0.023	0.65
CRP, mg/L	2.7 ± 3.5	2.2 ± 3.1	(− 0.6, 1.6)	0.322	0.30
Medication use, *n* (%)					
Infliximab	2 (17%)				
Adalimumab	2 (17%)				
Methotrexate	6 (50%)				
Leflunomide	1 (8%)				
Sulfasalazine	2 (17%)				
Tofacitinib	1 (8%)				
Hydroxychloroquine	4 (33%)				
NSAID	8 (67%)				
Prednisone	2 (17%)				

*Abbreviations: HIIT* High-intensity interval training, *HbA1c* Hemoglobin A1c, *BMI* Body mass index, *HAQ-DI* Health Assessment Questionnaire Disability Index, *DAS28* Disease Activity Score in 28 joints, *ESR* Erythrocyte sedimentation rate, *CRP* C-reactive protein, *NSAID* Nonsteroidal anti-inflammatory drug

Data are mean ± SD

### HIIT effects on cardiorespiratory fitness and physical function

Table 2 shows that 10 weeks of HIIT resulted in a 9 ± 4% increase in both relative (*p* < 0.001) and absolute (*p* < 0.001) cardiorespiratory fitness (range, 0–15%), similar to our previous observations with HIIT in younger healthy adults [25]. Body composition, including body mass index (BMI) and body fat percentage, were unchanged following training (both *p* > 0.05). Because physical functioning is generally impaired in patients with RA, we sought to determine function in a number of ways. Assessment of habitual physical activity was determined by the IPEQ and the SBAS. The IPEQ (*p* = 0.788) and SBAS (*p* = 0.096) results suggested that participants were no more active outside the prescribed training program than in it. As expected, because the training used a walking protocol, grip strength was unchanged following training (*p* > 0.05). However, there were significant improvements in lower limb function, as indicated by an 8 ± 4% reduction in time needed to walk 400 m (*p* = 0.001) and an 11 ± 12% increase in the number of chair-stands completed over 30 seconds (*p* = 0.035).

**Table 2:** Before and after high-intensity interval training values, CIs, and effect sizes for cardiorespiratory fitness and physical function

	Pre-HIIT	Post-HIIT	95% CI (lower, upper)	*p* Value	Effect size (*d*)
Cardiorespiratory fitness					
VO_2peak_, L/min	1.75 ± 0.4	1.89 ± 0.4	(− 3.0, − 1.8)	< 0.001	2.86
VO_2peak_, ml/kg/min	25.0 ± 6.6	27.1 ± 7.0	(− 0.2, − 0.13)	< 0.001	3.19
Physical function					
Physical activity questionnaires					
IPEQ	30.4 ± 24.6	28.8 ± 16.7	(− 11.2, 14.4)	0.788	0.08
SBAS	2.5 ± 0.5	2.9 ± 1.0	(− 0.9, 0.1)	0.096	0.52
Grip strength, kg	18.3 ± 7.2	19.0 ± 8.1	(− 1.7, 0.3)	0.166	0.43
Berg Balance Score	54.4 ± 4.0	54.7 ± 3.2	(− 1.2, 0.7)	0.555	0.18
TUG, s	8.8 ± 1.8	8.4 ± 1.9	(− 0.1, 0.1)	0.084	0.54
400-m walk test, s	251 ± 62	233 ± 51	(9.0, 27.4)	0.001	1.30
30-s chair stands, *n*	14 ± 4	17 ± 5	(− 4.0, − 0.2)	0.035	0.70

*Abbreviations: HIIT* High-intensity interval training, *IPEQ* Incidental and Planned Exercise Questionnaire, *SBAS* Stanford Brief Activity Survey, *TUG* Timed Up and Go, VO_2peak_ Aerobic capacity

Data are mean ± SD

### HIIT effects on systemic inflammation, white blood cell counts and metabolic health

In order to determine if exercise training had any effect on systemic inflammation and measures of glucose and fatty acid control, we assessed a small panel of pro- and anti-inflammatory markers, white blood cell counts, glucose, insulin, and nonesterified fatty acid (NEFA) concentrations, (Table 3). No changes were observed for IL-1β, IL-6, CXCL-8, TNF-α, or the anti-inflammatory cytokine IL-10 (all *p* > 0.05). There were no training-induced changes in complete blood cell count 16–24 hours after the last exercise bout, suggesting minimal to no influence of the final acute bout (all *p* > 0.05). Similarly, fasting glucose, insulin, and NEFAs did not change (all *p* > 0.05).

**Table 3:** Before and after high-intensity interval training values, CIs, and effect sizes for systemic inflammation, white blood cell counts, glucose, insulin and insulin resistance, and nonesterified fatty acid concentrations

	Pre-HIIT	Post-HIIT	95% CI (lower, upper)	*p* Value	Effect size (*d*)
Systemic inflammation					
IL-1β, pg/ml	0.12 ± 0.13	0.12 ± 0.11	(− 0.04, 0.06)	0.788	0.07
IL-6, pg/ml	1.06 ± 0.89	1.43 ± 2.02	(− 1.6, 0.8)	0.519	0.19
CXCL-8, pg/ml	9.69 ± 6.97	10.3 ± 10.0	(− 3.3, 2.2)	0.655	0.13
IL-10, pg/ml	0.5 ± 0.4	0.5 ± 0.3	(− 0.1, 0.1)	0.912	0.03
TNF-α, pg/ml	2.21 ± 0.96	2.31 ± 0.96	(− 0.5, 0.3)	0.570	0.17
White blood cells, ×10^6^/L					
Total count	5.6 ± 1.1	5.8 ± 1.2	(− 0.6, 0.2)	0.322	0.32
Neutrophils	2.8 ± 0.8	2.9 ± 0.9	(− 0.5, 0.1)	0.217	0.40
Lymphocytes	2.0 ± 0.6	2.1 ± 0.6	(− 0.4, 0.2)	0.623	0.15
Monocytes	0.6 ± 0.2	0.6 ± 0.2	(− 0.1, 0.1)	0.676	0.12
Eosinophils	0.3 ± 0.3	0.2 ± 0.1	(− 0.02, 0.2)	0.108	0.53
Neutrophils: lymphocytes	1.5 ± 0.6	1.5 ± 0.5	(− 0.4, 0.3)	0.810	0.07
Glucose, insulin, and NEFA					
Fasting glucose (mg/dl)	90.9 ± 7.7	93.1 ± 8.3	(− 4.8, 0.5)	0.098	0.55
Fasting insulin, μIU/ml	13 ± 4.9	12 ± 4.8	(− 0.8, 2.7)	0.264	0.34
HOMA-IR	1.67 ± 0.64	1.56 ± 0.62	(− 0.1, 0.3)	0.258	0.34
NEFA, mmol/L	0.55 ± 0.37	0.60 ± 0.36	(− 0.2, 0.1)	0.412	0.24

*Abbreviations: HIIT* High-intensity interval training, *IL* Interleukin, *CXCL* Chemokine C-X-C motif ligand, *TNF-α* Tumor necrosis factor-α, *HOMA-IR* Homeostatic model of assessment of insulin resistance, *NEFA* Nonesterified fatty acids

Data are mean ± SD

#### HIIT effects on innate immune function

##### Improved neutrophil migration and bactericidal function

**Fig. 1:**
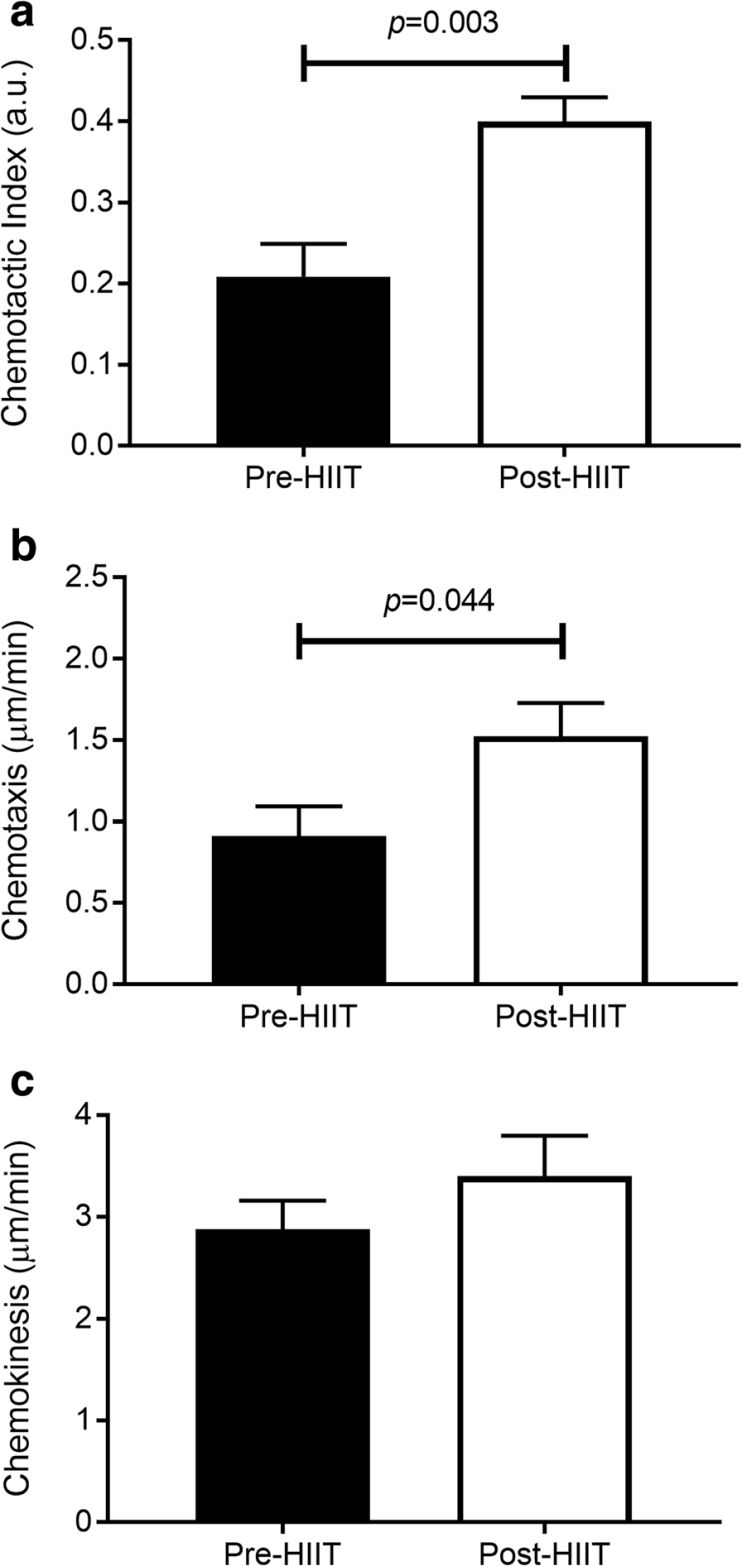
Neutrophil migratory changes following high-intensity interval training (HIIT). Neutrophils were migrated toward chemokine (C-X-C motif) ligand CXCL-8 and assessed pre- and post-HIIT for 10 weeks for overall chemotactic accuracy, known as the chemotactic index (**a**); directional speed, known as chemotaxis (**b**); and overall speed, known as chemokinesis (**c**). All data are mean ± SEM (*n* = 12)

**Fig. 2:**
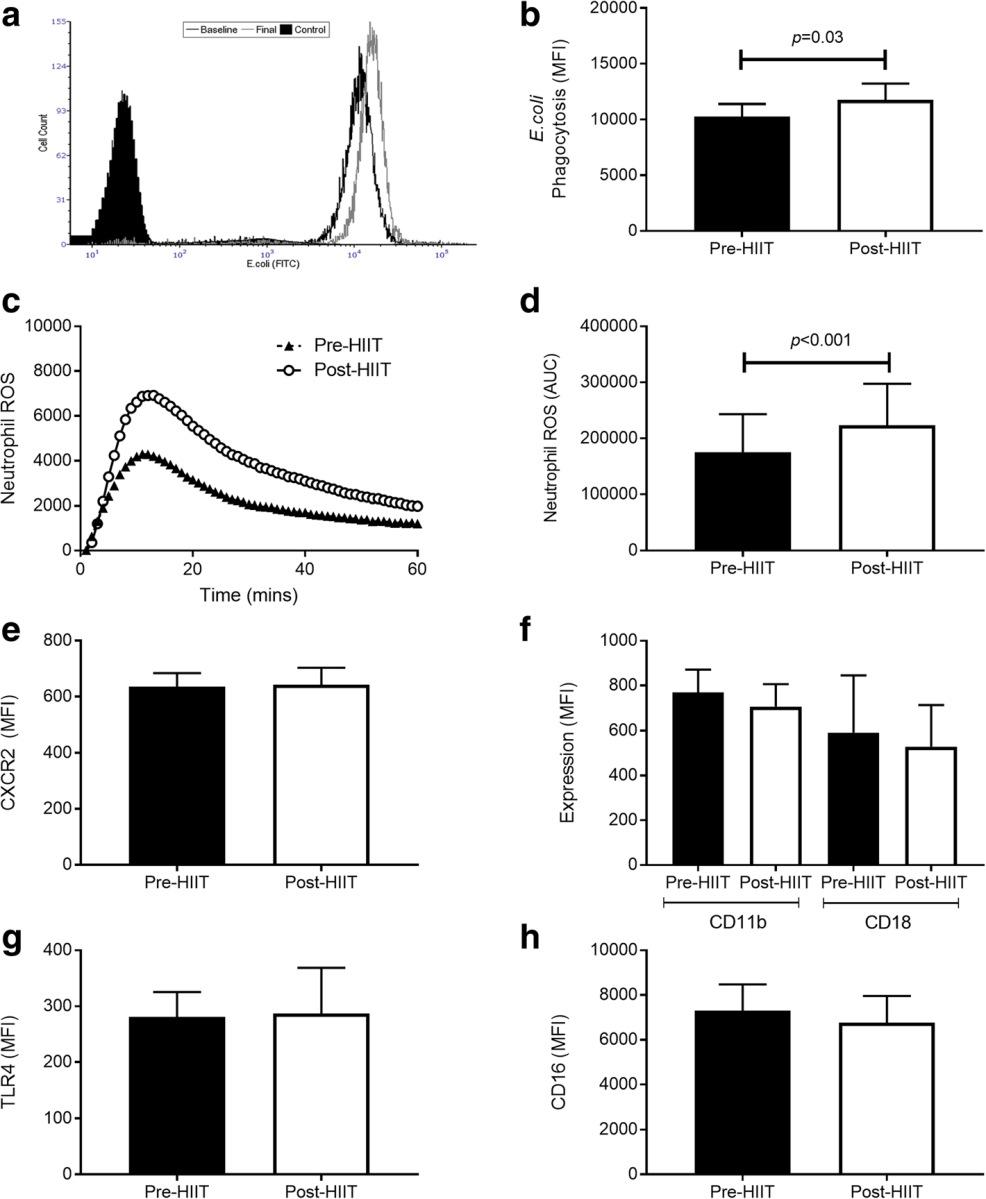
Neutrophil changes in response to 10 weeks of high-intensity interval training (HIIT). Representative flow cytometric histograms showing (**a**) improved neutrophil phagocytosis of *Escherichia coli*, (**b**) phagocytic capacity, (**c**) representative diagram of the kinetic phorbol 12-myristate 13-acetate-stimulated ROS production, and (**d**) ROS generation (AUC) and neutrophil surface receptor expression pre- and post-HIIT training for (**e**) CXC chemokine receptor 2 (CXCR2), (**f**) cluster of differentiation 11b (CD11b)/CD18, (**g**) Toll-like receptor 4 (TLR4), and (**h**) CD16. All data are mean ± SEM (*n* = 12). *MFI* Median fluorescence intensity

In response to 10 weeks of HIIT, migration of isolated RA peripheral blood neutrophils toward the chemokine CXCL-8 improved. A doubling of the chemotactic accuracy (chemotactic index; *p* = 0.003; CI, − 0.3, − 0.1; *d* = 1.1) was driven by improved directional speed (chemotaxis, *p* = 0.044; CI, − 1.2, − 0.02; *d* = 0.66), with no effect on overall speed (chemokinesis, *p* = 0.272; CI, − 1.5, 0.5; *d* = 0.33) (Fig. 1a–c). Following HIIT, neutrophils phagocytized more FITC-labeled *E. coli* (Fig. 2a and b) (*p* = 0.03; CI, − 2884, − 87; *d* = 0.71). Accompanying the improved phagocytosis, training enhanced neutrophil PMA-stimulated ROS production (Fig. 2d) (*p* < 0.001; CI, − 69,964, − 25,737; *d* = 1.4). No differences were observed for expression of the relevant surface receptors, which could account for these changes, namely the CXCL-8 receptor CXCR2 (Fig. 2e) (*p* = 0.865; CI, − 105, 90; *d* = 0.06), the ICAM-1 receptors CD11b/CD18 (Fig. 2f) (*p* = 0.404; CI, − 96, 207; *d* = 0.27), TLR4 (Fig. 2g) (*p* = 0.929; CI, − 158, 147; *d* = 0.03), or the Fcγ receptor CD16 (Fig. 2h) (*p* = 0.300; CI, − 571, 1652; *d* = 0.35). Taken together, these data suggest that HIIT improves neutrophil migratory accuracy and bactericidal functions in patients with stable RA.

##### Shifts from pro- to anti-inflammatory monocyte phenotypes

**Fig. 3:**
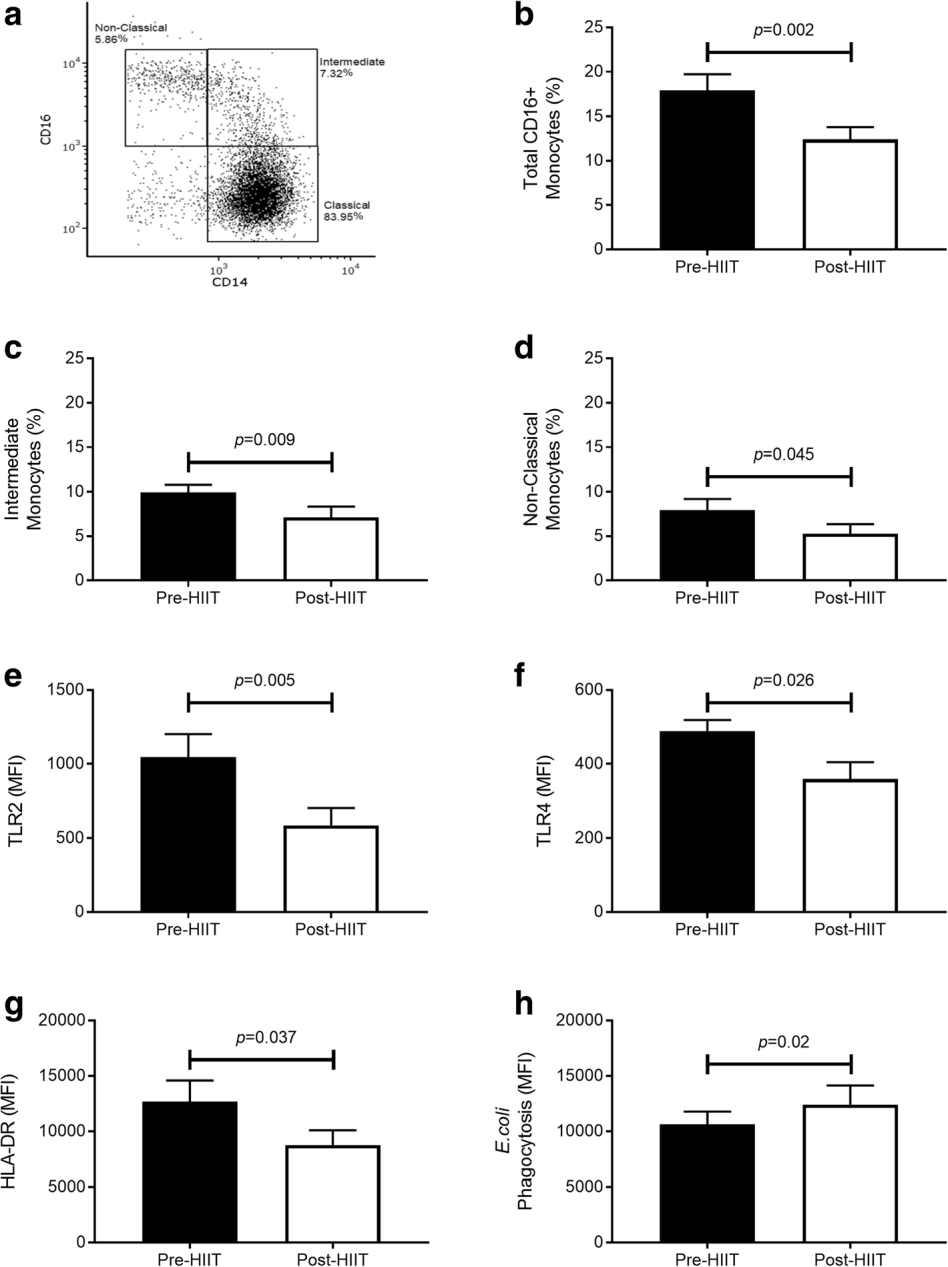
Monocyte changes in response to 10 weeks of high-intensity interval training (HIIT). **a** Representative forward vs. side scatter of cluster of differentiation 16 (CD16) expression on CD14^+^ monocytes. Frequency of (**b**) total CD16-expressing monocytes, (**c**) intermediate monocytes, and (**d**) nonclassical monocytes pre- and post-HIIT training. Surface expression of (**e**) Toll-like receptor 2 (TLR2), (**f**) TLR4, and (**g**) human leukocyte antigen (HLA)-DR on intermediate monocytes pre- and post-HIIT training. **h** Phagocytosis of fluorescein isothiocyanate-labeled *Escherichia coli* pre- and post-HIIT training. All data are mean ± SEM (*n* = 12). *MFI* Median fluorescence intensity

Depending on their expression of CD14 and CD16, monocytes can be considered to be composed of three distinct populations (Fig. 3a). Following 10 weeks of HIIT, there were significant reductions in the frequency of total CD16-expressing proinflammatory monocytes (Fig. 3b) (*p* = 0.002; CI, 2.5, 8.4; *d* = 1.3) owing to reduced frequency of both intermediate CD14^bright^/CD16^positive^ (Fig. 3c) (*p* = 0.009; CI, 0.9, 4.7; *d* = 1.04) and nonclassical CD14^dim^/CD16^bright^ (Fig. 3d) (*p* = 0.045; CI, 0.1, 5.3; *d* = 0.73) monocytes. On intermediate monocytes, there was reduced expression of TLR2 (Fig. 3e) (*p* = 0.005; CI, 182, 738; *d* = 1.27), TLR4 (Fig. 3f) (*p* = 0.026; CI, 20, 239; *d* = 0.90) and HLA-DR (Fig. 3g) (*p* = 0.037; CI, 306, 7528; *d* = 0.83). On classical CD14^bright^/CD16^negative^ and nonclassical monocytes, there were no differences for TLR2, TLR4, or HLA-DR expression. Additionally, there was a trend toward an increased percentage of classical monocytes at the expense of proinflammatory, CD16^+^ monocytes (*p* = 0.062; CI, − 5.7, 0.2; *d* = 0.68). A greater percentage of classical monocytes may have accounted for increased phagocytosis of FITC-labeled *E. coli* following training (Fig. 3h) (*p* = 0.02; CI, − 3260, − 340; *d* = 0.83). Taken together, these data suggest that HIIT improves the balance of inflammatory pathologically related CD16-expressing monocytes to CD16 negative monocytes.

#### Correlations on disease activity, fitness, body composition, immune function, and inflammation

To determine relationships between changes in our outcome variables, we conducted correlation analyses between changes in measures of disease activity, fitness, body composition, immune function, and inflammatory markers. The only significant correlation observed was between changes in relative VO_2peak_ and IL-10, with increasing fitness associated with higher concentrations of IL-10 (rho = 0.653; *p* = 0.029).

## Discussion

Ten weeks of high-intensity interval walking above exercise intensity health guidelines was associated with reduced disease activity, increased aerobic capacity, and improved antibacterial innate immune function in patients with RA. Without a sufficient control group, it is unclear exactly how much HIIT walking contributed to these changes. Changes in disease activity were associated with reductions in ESR and joint swelling and with increased global perceived health. Furthermore, there were associations with changes in innate immune function, including neutrophil chemotaxis, bacterial phagocytosis and ROS production, improved monocyte bacterial phagocytic capacity, and a shift toward a classical CD14^bright^/CD16^negative^ monocyte phenotype. These functional immune improvements potentially enhance antibacterial function, reducing the risk of infection.

### Disease activity, physical fitness, and function in RA

There is a significant body of literature showing that physical activity and exercise training are safe for patients with RA and do not aggravate disease activity [47–50]. Although studies have suggested that individual components of disease activity can be improved, few have reported actual lowering of disease activity scores [48]. In the present study, using a composite, well-validated disease activity score including assessment of 28 joints for tenderness and swelling, perceived global health, and inflammatory markers (ESR or CRP), overall disease activity was reduced by 38% following training. Reductions appeared to be driven primarily by improvements in joint health, as determined by swollen and tender joints, improved participant feelings of health, and reduced ESR. This reduction was sufficient to reclassify participants on average from moderate to low disease activity. Such a reduction is unlikely to be observed without exercise training; however, because we did not have a control group, this cannot be discounted.

It is not completely clear what accounted for our observed disease activity improvements. One possibility is that by using higher intensities and grade elevations, HIIT had effects on lower extremity strength. Strength improvements were evident by improved sit-to-stand ability. Walking was at faster speeds than individual participants’ normal speeds, as evidenced by comparisons with calculated speeds from both the TUG and 400-m walk speed (data not shown) tests. As such, the only other exercise intervention studies to show disease score reductions incorporated resistance training, suggesting a link between muscle strength and disease activity [51–55]. In light of this, we now add to the literature that HIIT walking is likely associated with improved disease activity. Further, as compared with other aerobic exercise interventions, no significant adverse events and a 9% increase in cardiorespiratory fitness were observed, suggesting that our program is safe and effective in improving cardiorespiratory fitness [30, 32].

### HIIT and immune function in RA

Although RA is considered an inflammatory disease characterized by increased systemic and joint inflammation, the pathology of the inflammation is due primarily to a deregulated immune system. Specifically, there is a growing body of evidence suggesting that habitual physical activity and exercise training promote enhanced immune function in both adaptive and innate cells [56]. Although exercise can improve immune function in age-comparable healthy adults [57], few studies have assessed the effects of exercise training on immune function in patients with RA. Because patients with RA are at increased risk of opportunistic infections owing to impaired and reduced immune responses, promoting better immune function likely reduces these risks. Very few studies have assessed immune responses to exercise in RA, with no changes in lymphocyte proliferation or natural killer (NK) cell tumor cell cytotoxicity [31, 58], suggesting that exercise may not enhance primary cell functions. However, because patients with RA are at increased risk of infection and require optimal neutrophil and monocyte function, we were interested in whether HIIT would improve antibacterial functions of innate immune cells.

When compared with healthy age-matched control subjects, patients with stable RA have similar neutrophil migration [59, 60]. Although increased neutrophil migration from the blood to the synovium is observed in the early stages of RA, improved chemotactic accuracy should benefit older patients with RA [4, 59, 61], specifically because poor chemotactic accuracy results in more aberrant tissue damage and elongated resolution of infection and increased inflammatory insult. In a study of healthy older adults, those who completed more steps per day (10,370 ± 3221 vs. 4746 ± 1351) had better neutrophil chemotactic accuracy, suggesting that exercise training may improve neutrophil migratory capacity [20]. Neutrophil migration was also improved in women with hypertension older than 60 years of age following 6 months of exercise training and in young sedentary participants following 2 months of cycling training [62, 63]. We add to the literature by showing, for the first time to our knowledge, that HIIT is associated with improved neutrophil migration toward a common chemokine produced during infectious episodes in older adults with stable RA.

Along with migration, other critical primary neutrophil functions include phagocytosis and ROS killing of pathogens. Although excessive ROS production in the synovium contributes to disease pathology, it is unclear what role blood neutrophil ROS play in RA [5, 64]. Compared with healthy control subjects, in RA blood neutrophil ROS are either similar or reduced [5, 7], whereas reduced ROS with aging is a classic hallmark of neutrophil dysfunction [65]. Similarly, there is no clear consensus regarding whether peripheral blood neutrophil phagocytosis is altered in RA [66–70]. Paino and colleagues suggested that phagocytosis of *E. coli* is greater in older women with RA than in matched control subjects [67], whereas Turner and colleagues suggested that phagocytosis is reduced in RA [68]. Similarly to our previous HIIT study in healthy adults [25], we show increased stimulated ROS production and increased bacterial phagocytosis following exercise training, suggesting an enhanced ability of neutrophils to kill bacterial pathogens. Furthermore, in agreement with previous studies, functional improvements are not associated with changes in relevant cell surface receptor expression, suggesting intrinsic cellular changes [4, 20, 25].

Neutrophil dysfunction is associated with increased infection risk, and so is monocyte/macrophage dysfunction. Further, with RA and aging, monocytes adopt more of an inflammatory phenotype with the potential to add to the inflammatory milieu. There are three subtypes of blood monocytes with distinct inflammatory and functional characteristics dependent on their expression of CD16 [71–73]. In addition to CD16, TLR2 and TLR4 are typically expressed more on monocytes/macrophages in RA [10, 11, 74]. In healthy sedentary adults, HIIT can reduce expression of CD16, TLR2, and TLR4 while increasing monocyte phagocytosis of *E. coli* [25, 28]. We add that in older patients with stable RA, HIIT is associated with increased phagocytic capacity of *E. coli* as well as reductions in the total percentage of CD16-expressing monocytes through reductions in the percentage of both intermediate and nonclassical monocytes. Furthermore, the expression of TLR2, TLR4, and HLA-DR was reduced on the intermediate proinflammatory monocytes, suggesting a reduced proinflammatory phenotype.

### HIIT and inflammation in RA

RA medications are aimed at controlling inflammation [75]. Whether exercise training can reduce systemic inflammation in RA is an open question [18–20, 76]. It is likely that exercise has clinically or pathologically meaningful anti-inflammatory effects primarily in individuals with high, uncontrolled inflammation or when exercise is associated with reductions in body fat. The anti-inflammatory effects of exercise training have been reviewed extensively; yet, the mechanisms are still unclear. Long-term training likely exerts most of its anti-inflammatory effects by changing adipocyte mass and function [18]. In the present study, participants had well-controlled stable RA, were taking a range of anti-inflammatory medications, and had minimal nonsignificant changes in body fat; thus, we were not surprised to observe a lack of meaningful changes in systemic concentrations of CRP, IL-1β, IL-6, CXCL-8, IL-10, or TNF-α. These findings are similar to other aerobic-based exercise programs, suggesting that aerobic training without weight loss is insufficient to reduce most inflammatory markers in patients with RA, even those with well-controlled RA [30]. However, in agreement with studies using resistance training, we observed a significant reduction in the inflammatory marker ESR [51]. It is unclear on the basis of our study whether the changes in joint health, as determined by swollen and tender joint count reductions, were associated with reduced joint-specific inflammation. It is possible that joint health improved because of fluid shifts induced by mechanical loading; however, a number of the healthy joints were observed in the hands and arms. To determine relationships with training and inflammation, we conducted correlations between changes in fitness, body fat, and the aforementioned cytokines. No relationships were observed, other than increases in peak VO_2_ being associated with increasing concentrations of the anti-inflammatory cytokine IL-10. Future work aimed at understanding relationships between systemic and joint inflammation and exercise training may help illuminate this finding.

### Limitations and future directions

Our study has limitations. A lack of an age-matched healthy control group does not allow us to state whether immune function was altered in our study group as a result of their disease. However, a control group in such a population is difficult to select because patients with RA are often more sedentary than healthy control subjects, their immune system shows signs of premature aging, and immune-modifying medications complicate immune function comparisons. In light of this, we could have recruited an RA nonexercise control group in order to show that changes were in fact due to the intervention and not dependent on time. However, a previous exercise study with an RA control group showed that 8 weeks of training was sufficient to increase cardiorespiratory fitness in the exercise group only. Subsequently, the control group did not have changes in immune cell proliferation, NK cell cytotoxicity, or inflammatory cytokine concentrations, suggesting that time alone does not change fitness or alter the immune system and inflammation in patients with RA [31]. Therefore, we are confident that the changes we observed were due to the exercise intervention and not to time alone.

Although this study was a pilot, considerations should be acknowledged regarding the population studied as well as the generalizability and feasibility of a larger, broader RA population to complete such an intervention. Our participants, although representative of our local RA population, were younger and had lower disease activity than much of the larger national RA demographics. As such, it is unclear whether such an intervention would be feasible and provide similar results in an older group with higher disease activity. Further, this was a supervised, structured exercise intervention, which would limit many individuals who are unable to access such expertise and gym facilities. Future studies should address a broader RA population and determine whether this program is feasible and can be transferred to a community- or home-based setting.

Further, we are confident that a significant portion of the effects was due to the intervention and that participants were not engaging in extra exercise outside our study design. Hower, we cannot be certain this did not occur. Although we used validated questionnaires, compared to quantifiable accelerometer data, questionnaires have inherent reliability issues. 

The mechanisms by which exercise training in RA modifies disease activity and joint health or how changes in immune function can contribute are unclear. There is the potential that exercise training can improve immune-mediated joint health by moving dysfunctional cells out of the joint, allowing for replacement with healthier immune cells. Although this hypothesis remains untested in RA, animal exercise models suggest that exercise is associated with a redistribution of immune cells between tissues [77, 78]. Therefore, to elucidate the mechanisms associated with joint improvements, disease activity, and immune function, researchers in future studies should try to compare systemic exercise effects with those in the synovial fluid and local joint tissue.

## Conclusions

We observed, for the first time to our knowledge, that 10 weeks of high-intensity interval walking in patients with stable RA was associated with clinically meaningful reductions in disease activity. Improvements in disease activity were accompanied by changes in immune cell function indicative of improved innate immunity and reduced risk of opportunistic bacterial infection. Our data suggest that HIIT walking could be an efficient, tolerable, and highly effective intervention to augment disease activity and improve overall health in patients with RA.

## Abbreviations

APC: Allophycocyanin
BMI: Body mass index
BSA: Bovine serum albumin
CD: Cluster of differentiation
CRP: C-reactive protein
CXCL: Chemokine (C-X-C motif) ligand
CXCR: CXC chemokine receptor
DAS28: Disease Activity Score in 28 joints
ELISA: Enzyme-linked immunosorbent assay
ESR: Erythrocyte sedimentation rate
FITC: Fluorescein isothiocyanate
HAQ-DI: Health Assessment Questionnaire Disability Index
HADS: Hospital Anxiety and Depression Scale
HbA1c: Hemoglobin A1c
HBSS: Hanks’ balanced salt solution
HIIT: High-Intensity Interval Training
HLA-DR: Human leukocyte antigen-DR
HOMA-IR: Homeostasis model assessment of insulin resistance
ICAM-1: Intercellular adhesion molecule 1
IL: Interleukin
IPEQ: Incidental and Planned Exercise Questionnaire
LLOD: Lower limit of detection
MFI: Median fluorescence intensity
NEFA: Nonesterified fatty acids
NK: Natural killer cell
PBMC: Peripheral blood mononuclear cell
PE: Phycoerythrin
PI: Propidium iodide
PMA: Phorbol 12-myristate 13-acetate
RA: Rheumatoid arthritis
RER: Respiratory exchange ratio
RPMI: Roswell Park Memorial Institute
SBAS: Stanford Brief Activity Survey
TLR: Toll-like receptor
TNF: Tumor necrosis factor
TUG: Timed Up and Go
VAS: Visual analogue scale for pain
VO_2peak_: Aerobic capacity
